# Notoginsenoside Fc Accelerates Reendothelialization following Vascular Injury in Diabetic Rats by Promoting Endothelial Cell Autophagy

**DOI:** 10.1155/2019/9696521

**Published:** 2019-09-03

**Authors:** Jingjing Liu, Chunyu Jiang, Xu Ma, Lishuai Feng, Jianbo Wang

**Affiliations:** Department of Interventional Radiology, The Sixth People's Hospital Affiliated to Shanghai Jiaotong University, Shanghai 200233, China

## Abstract

Interventional therapies, such as percutaneous transluminal angioplasty and endovascular stent implantation, are used widely for the treatment of diabetic peripheral vascular complications. Reendothelialization is an essential process in vascular injury following interventional therapy, and hyperglycemia in diabetes mellitus (DM) plays an important role in damaging endothelial layer integrity, leading to the retardance of reendothelialization and excessive neointimal formation. Notoginsenoside Fc (Fc), a novel saponin isolated from *Panax notoginseng*, effectively counteracts platelet aggregation. Nevertheless, the potential effects and molecular mechanisms of Fc on reendothelialization have yet to be explored. In this study, we present novel findings that show the benefit of Fc in accelerating reendothelialization and alleviating excessive neointimal formation following carotid artery injury in diabetic Sprague–Dawley rats *in vivo*. Simultaneously, the decreased autophagy of the injured carotid artery in diabetic rats was restored by Fc treatment. Our *in vitro* results also demonstrated that Fc promoted endothelial cell proliferation and migration under high-glucose treatment by increasing autophagy. In summary, this study supported the notion that Fc could accelerate reendothelialization following vascular injury in diabetic rats by promoting autophagy, suggesting that Fc may exert therapeutic benefits for early endothelial injury and restenosis following intervention in diabetes-associated vascular diseases.

## 1. Introduction

Over 300,000,000 individuals have diabetes mellitus (DM) worldwide [1], and approximately 60–80% of DM patients present with vascular complications [2]. At present, interventional therapies, such as percutaneous transluminal angioplasty and endovascular stent implantation, have been widely used for the treatment of diabetic peripheral vascular complications and have shown promising results in clinical practice [3, 4]. However, vascular restenosis following intervention remains a serious problem in angioplasty treatment for diabetic peripheral artery diseases and the restenosis rate can reach as high as 50–70% [5].The main reason for this effect is that the drug-eluting stent or balloon not only inhibits the proliferation of smooth muscle cells but also inhibits reendothelialization, which is essential for the prevention of excessive neointima [6]. Therefore, new strategies for efficient reendothelialization are needed. Notoginsenoside Fc (Fc), a novel protopanaxadiol- (PPD-) type saponin isolated from the leaves of *Panax notoginseng*, effectively counteracts platelet aggregation [7]. There are multiple reports implicating other saponins derived from P. notoginseng in cardiovascular pathophysiology [8]. Dammarane-type triterpene saponins, including ginsenosides and notoginsenosides, contributed to the abovementioned pharmacological effects, and these saponins can be divided into PPD and protopanaxatriol (PPT) types based on their aglycone skeleton [9]. It has been demonstrated that Fc exhibits various pharmacological activities as a folk medicine or dietary supplement [10, 11]. However, little information regarding the effects of Fc on reendothelialization and the underlying mechanism is available.

Autophagy is an evolutionarily conserved catabolic process that mediates protein degradation, organelle turnover, and recycling of cytoplasmic contents in a lysosome-dependent manner [12]. Recent studies have shown that autophagic dysregulation is associated with several cardiovascular pathological processes, such as atherosclerosis, cardiac hypertrophy, and cardiomyopathies [13, 14]. Uberti et al. reported that vitamin D-activated autophagy promotes human umbilical vein endothelial cell survival under oxidative stress, suggesting that autophagy represents a target for endothelial cell protection [15]. Endothelial apoptosis interferes with endothelial regeneration and function, thus retarding the endothelial wound healing process [16]. Moreover, Ye et al. indicated that knockdown of Beclin 1, a key component in autophagy initiation, exacerbated neointimal formation after rat carotid injury, which was associated with retarded reendothelialization [17]. In addition, many studies have demonstrated that *P*. *notoginseng* protects many types of cells via autophagy induction [18–20]. Therefore, in the present study, we investigated whether Fc protects against endothelial cell injury, accelerates reendothelialization, and attenuates excessive neointimal formation in DM rats via autophagy induction.

With this information, the present study is aimed at determining whether Fc accelerates reendothelialization and alleviates excessive neointimal formation following carotid artery injury in diabetic rats. We hypothesized that the underlying mechanism involves promoting autophagy in rat aortic endothelial cells (RAOECs).

## 2. Materials and Methods

### 2.1. Drug Preparation

**Figure 1:**
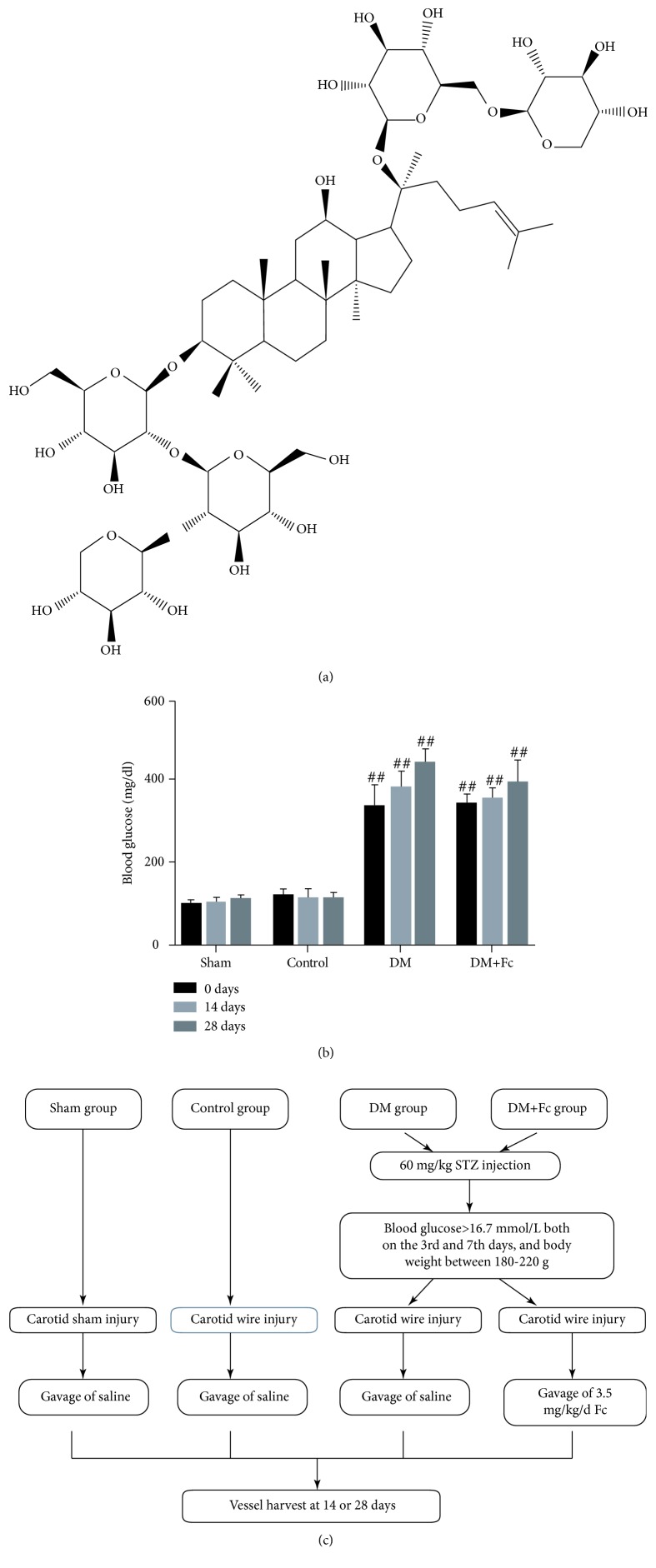
(a) Molecular structure of notoginsenoside Fc (Fc). (b) Blood glucose levels in the different rat groups at days 14 and 28. DM represents diabetes mellitus. (c) A schematic diagram illustrating the experimental animal groups and different treatments. STZ represents streptozotocin.

Notoginsenoside Fc (chemical structure C_58_H_98_O_26_, molecular weight = 1211.4 Da, and purity ≥ 98%) was purchased from Shanghai Yuanye Bio-Technology Co. Ltd. (Shanghai, China). The molecular structure is shown in Figure 1(a).

### 2.2. Animal Preparation

Animals and forage were purchased from the Model Animal Research Centre of Nanjing University (Jiangsu, China). This study conformed to the Guide for the Care and Use of Laboratory Animals published by the US National Institutes of Health (NIH publication no. 85-23, revised 1996), and the Institutional Animal Care and Use Committee of Shanghai Sixth People's Hospital approved the protocol. All rats were housed in individually ventilated cages (three or four per cage) under specific pathogen-free conditions. Housing was temperature controlled, with a 12 h/12 h light/dark cycle. In total, 48 male Sprague–Dawley rats (200 ± 20 g) were randomly separated into four groups: sham group (*n* = 12), control group (*n* = 12), DM group (*n* = 12), and DM+Fc group (*n* = 12). After 12 h of fasting, the DM and DM+Fc groups were given an intraperitoneal injection of 60 mg/kg streptozotocin (STZ). Fasting blood samples were taken from the tail vein of rats and the blood glucose levels of all rats were tested twice on the days 3 and 7, respectively, after STZ injection. A Roche blood glucose meter and Roche test paper were used to measure fasting blood glucose levels. A fasting blood glucose > 16.7 mmol/L, both on day 3 and day 7, represented successful establishment of a diabetic rat model.

### 2.3. Carotid Artery Injury and Evans Blue Staining

After successful modeling, animals were fasted without water deprivation for 12 h before wire injury of the rat carotid artery was performed as described previously [21]. A 2-French balloon catheter (Edwards Lifesciences, Irvine, CA, USA) was inserted through the left external carotid artery into the common carotid artery and insufflated three times with 2 atm of pressure. Following injury, the external carotid artery was rapidly ligated and blood flow was resumed. Then, the DM+Fc group began drug treatment with a gavage of 3.5 mg/kg/d Fc until the rats were killed. The other three groups were given the same dose of saline. A schematic diagram to illustrate the different experimental animal groups and treatments is showed in Figure 1(c). The reendothelialization rate was confirmed by Evans blue dye (Sigma-Aldrich, St. Louis, MO, USA) staining. The area of remaining denudation at 14 and 28 days after injury was determined by left thigh femoral vein injection of 0.5% Evans blue dye after the rat was anesthetized. Subsequently, the tissue was cut longitudinally, washed with phosphate-buffered saline (PBS), and fixed with 4% paraformaldehyde. Samples were photographed using a dissecting microscope. Deendothelialized areas were defined as areas that stained blue, as assessed using ImageJ software (NIH, Bethesda, MD, USA). Simultaneously, the blood glucose of the rats was measured at days 0 (before carotid artery injury), 14, and 28 (Figure 1(b)).

### 2.4. Hematoxylin and Eosin (HE) Staining

On day 28 after injury, rat arteries were harvested and embedded in paraffin for HE staining. Each specimen was incubated in 4% paraformaldehyde for 24–48 h and then embedded in paraffin. Paraffin sections (3–5 mm thick) were dewaxed, stained, examined microscopically, and photographed. Images were captured using a fluorescence microscope (Jenoptik, Jena, Germany) and analyzed using ImageJ software. Neointimal and media areas were computed as follows: neointimal area = internal elastic lamina (IEL) area − lumen area; media area = external elastic lamina area − IEL area.

### 2.5. Immunohistochemistry

At 14 days after injury, rat arteries were harvested and embedded in optimal cutting temperature (OCT) compound (Tissue-Tek; Sakura Finetek, Torrance, CA, USA), snap-frozen in liquid nitrogen, and stored at −80°C for further use. Then, 7 *μ*m thick sections were cut at 500 mm intervals of the injured carotid artery (4 mm) and sections from the middle of the segments were stained with HE for immunohistochemistry. Samples were immunostained using a rabbit anti-Beclin 1 antibody (Cell Signaling Technology, Boston, MA, USA). The vessels were then stained with horse radish peroxidase-conjugated anti-rabbit IgG polymer and finally colored with 3,3-diaminobenzidin. Representative histological photomicrographs are shown.

### 2.6. Cell Culture

RAOECs were obtained from the American Type Culture Collection (Manassas, VA, USA). Cells were cultured in Dulbecco's modified Eagle's medium (DMEM) supplemented with 10% fetal bovine serum (Gibco, Carlsbad, CA, USA). RAOECs were cultivated in a humidified atmosphere at 37°C with 5% CO_2_. The growth medium was replaced every 2 days, and passages 4–6 were used for experiments. According to our previous experiments [22], the cells were divided into the following groups: (i) normal glucose (NG) group: cells were incubated in DMEM containing 5.6 mM D-glucose; (ii) NG+Fc group: cells were treated with DMEM and 20 *μ*M Fc for 24 h; (iii) high-glucose (HG) group: cells were treated with 30 mM D-glucose for 24 h; and (iv) HG+Fc group: cells were treated with 30 mM D-glucose and 20 *μ*M Fc for 24 h. In addition, cells were pretreated with 5 mM 3-methyladenine (3-MA) (Selleck Chemicals, Houston, TX, USA) for 2 h to inhibit autophagy.

### 2.7. Cell Proliferation Assay

RAOECs were seeded onto a 96-well plate with 1 × 10^4^ cells per well. After growing to 70–80% confluence, cells were treated with the corresponding drugs for 24 h. Cell cycle analyses were performed using the Cell Cycle Phase Determination Kit (Yeasen) according to the manufacturer's instructions. Each experiment was performed independently at least three times.

### 2.8. Wound Healing Assay

The cell wound healing assay was employed to measure cell migration. RAOECs were inoculated into 6-well plates (5 × 10^5^ cells/well) and grown for 24 h to reach 70–80% confluence. Following 12 h of serum withdrawal, three scratches were made in each well using a P-20 pipette. Cells were incubated for 24 h with the indicated treatment to enable migration, and images of each well were taken to measure gap changes in the scratches.

### 2.9. Monitoring Autophagy Flux Using the mRFP-eGFP-LC3B Plasmid

RAOECs were individually seeded onto 35 mm glass bottom dishes for confocal microscopy. When cells grew to 80% confluence, the original medium was discarded; 4 *μ*g of the mRFP-eGFP-LC3B plasmid (GenePharma, Shanghai, China) was dissolved in the Opti-MEM reduced serum medium and then mixed with 5 *μ*L of liposomes using Lipofectamine® 2000 Reagent (Invitrogen, Carlsbad, CA, USA). The mixture was added to the cells and replaced with serum medium after 4–6 h. Finally, cells were immobilized by 4% paraformaldehyde for 20 min and washed three times with PBS after 48 h. For nuclear staining, cells were stained with DAPI for 15 min in the dark. Samples were observed and photographed with a confocal microscope (LSM 710 META; Zeiss, Oberkochen, Germany). At least eight cells (per experiment) were randomly selected to determine the number of mRFP-LC3B or eGFR-LC3B puncta in each group, and the numbers of red and yellow puncta were counted as previously described [23].

### 2.10. Transmission Electron Microscopy

RAOECs were immobilized by glutaraldehyde in an atmosphere of 4°C. After ethanol dehydration and embedding in epoxy resin, ultrathin sections were prepared using an LKB2-III ultrathin microtome and stained with both uranyl acetate and lead citrate. Changes in the cellular ultrastructure were observed and photographed with an EM420 transmission electron microscope.

### 2.11. Reverse Transcription-Quantitative PCR (RT-qPCR)

Total RNA was extracted with TRIzol reagent (Invitrogen) from RAOECs. The concentration of the isolated RNA sample was measured using a NanoDrop 2000 (Thermo Fisher Scientific, Carlsbad, CA, USA) and then reverse transcribed to obtain complementary DNA using a PrimeScript RT Reagent Kit (TaKaRa, Dalian, China). PCR was then performed using SYBR Premix Ex Taq (TaKaRa) with LC3B-, Beclin 1-, p62-, and GAPDH-specific primers. The primer pairs were as follows: LC3B: 5′-TACGAGAGCGAGAGAGATGAA-3′ (forward) and 5′-GCCTTCAGAGCTGACATGTAT-3′ (reverse); Beclin 1: 5′-ATGCCCACTTCAGCATCTC-3′ (forward) and 5′-TCACTGTCTTCCTCCTCTACTT-3′ (reverse); p62: 5′-CTGATCCCTGTCAAGCAGTATC-3′ (forward) and 5′-AGATCTTTGCACCAGTCTCTTC-3′ (reverse); GAPDH (internal control): 5′-GCAAGGATACTGAGAGCAAGAG-3′ (forward) and 5′-GGATGGAATTGTGAGGGAGATG-3′ (reverse). Relative mRNA expression levels were normalized to GAPDH and are presented as 2^−ΔΔCt^ values.

### 2.12. Western Blot Analysis

The carotid arteries of rats or cells were lysed with cell lysis buffer (Beyotime Institute of Biotechnology) supplemented with 0.5 mM phenylmethanesulfonylfluoride. Following centrifugation (12,000 rpm, 15 min, 4°C), protein concentrations were determined using a Bicinchoninic Acid Protein Assay Kit (Beyotime Institute of Biotechnology). The same mass of total protein was separated by sodium dodecyl sulfate-polyacrylamide gel electrophoresis and transferred to polyvinylidene difluoride membranes (0.22; Millipore, Billerica, MA, USA). Membranes were blocked with 5% nonfat milk in Tris-buffered saline containing 0.1% Tween 20 for 2 h at room temperature. Membrane-bound proteins were probed with the indicated primary antibody (1 : 500–1 : 1,000 dilution) overnight at 4°C, followed by horseradish peroxidase-conjugated secondary antibodies (1 : 5,000; Proteintech, Chicago, IL, USA) at room temperature for 1 h. Protein bands were detected with an enhanced chemiluminescence detection kit (Cell Signaling Technology) and photographed using a GE Amersham Imager 600 imaging system (GE Life Sciences, Chicago, IL, USA). Antibodies against LC3B (cat. no. 2775S), p62 (cat. no. 5114S), Beclin 1 (cat. no. 3738S), PCNA (cat. no. 13110S), and *β*-actin (cat. no. 4970S) were purchased from Cell Signaling Technology.

### 2.13. Statistical Analysis

All data were from at least three independent experiments and are presented as the means ± standard deviation. Statistical analyses involving multiple groups were performed by one-way analysis of variance followed by the least significant difference test using SPSS software (ver. 20.0; IBM Corp., Armonk, NY, USA). *P* < 0.05 was considered statistically significant.

## 3. Results

### 3.1. Fc Accelerates Reendothelialization following Carotid Artery Injury in Diabetic Sprague–Dawley Rats

**Figure 2:**
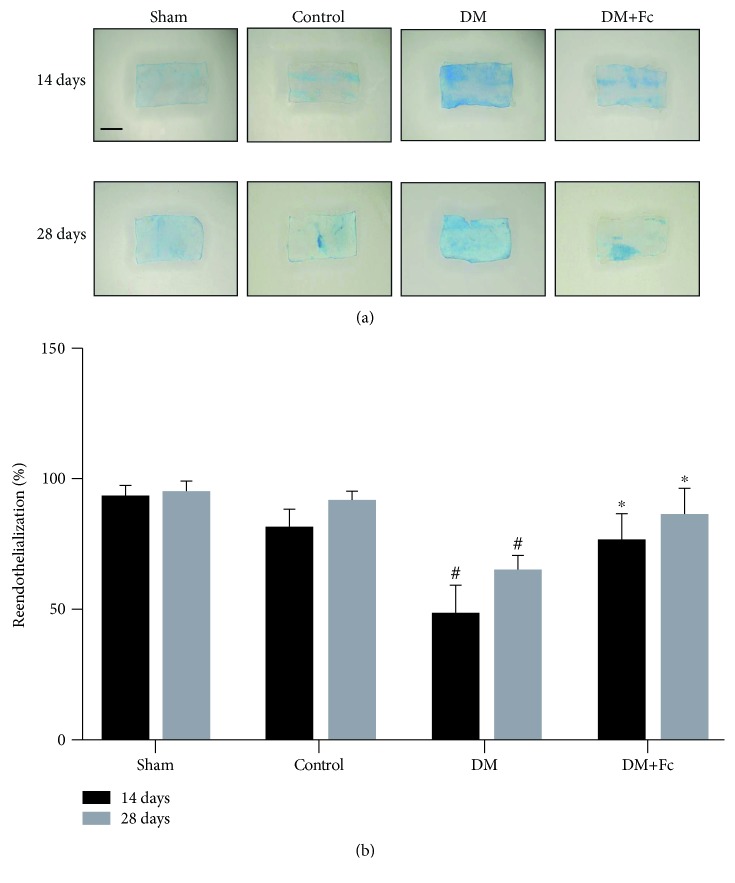
Effects of notoginsenoside Fc (Fc) on reendothelialization following carotid artery injury. Reendothelialization was quantified in Evans blue-stained carotid arteries at 14 and 28 days after vascular wire injury. (a) Blue staining indicates endothelial denudation. (b) The reendothelialization rates in the rats across treatment groups were analyzed statistically. The images are at 10x magnification. Scale bar = 1 mm. Data are shown as the mean ± standard deviation. ^#^*P* < 0.05 and ^##^*P* < 0.01 vs. the control group; ^∗^*P* < 0.05 vs. the diabetes mellitus (DM) group. *n* = 6.

To explore the effects of Fc on endothelial repair ability following vascular wire injury *in vivo*, we used Evans blue staining to examine reendothelialization of the carotid arteries in rats. As shown in Figure 2, the endothelium was gradually regenerated after injury and the reendothelialization rate reached 80.5 ± 5.8% by day 14 and 90.2 ± 3.5% by day 28, identical to noninjured sham rats. In diabetic rats, the reendothelialization rate was only 47.8 ± 9.2% by day 14 and 64.4 ± 4.8% by day 28, which was significantly retarded compared to the injured control. However, the repair capability of the injured artery was markedly improved by treatment with Fc. In the DM+Fc group, the reendothelialization rate was 75.9 ± 8.7% by day 14 and 85 ± 9.3% by day 28.

### 3.2. Fc Retards Excessive Neointimal Formation following Carotid Artery Injury in Diabetic Sprague–Dawley Rats

**Figure 3:**
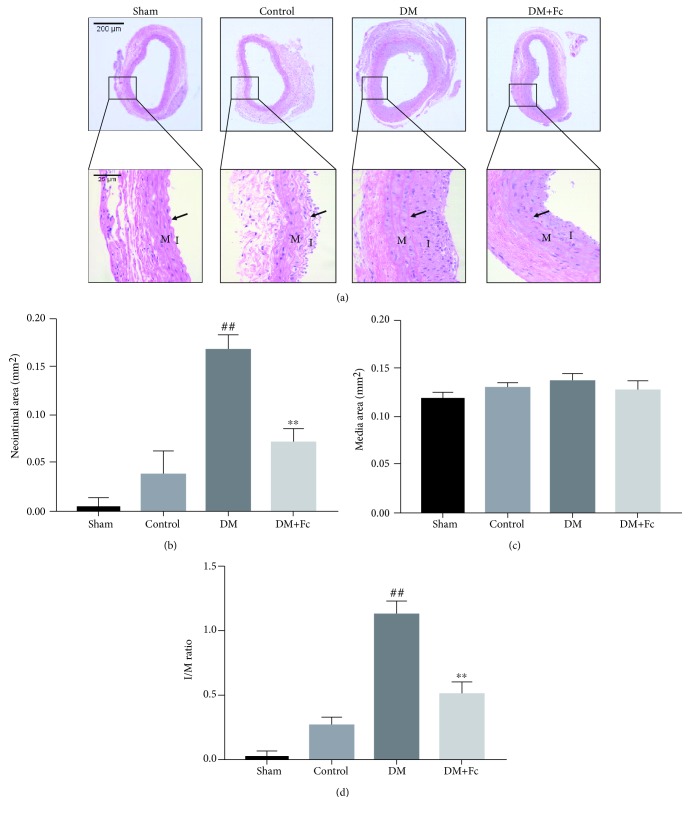
Effects of notoginsenoside Fc (Fc) on pathomorphology and intimal hyperplasia following carotid artery injury. (a) Representative photographs of carotid arteries by hematoxylin-eosin staining at 14 days are shown. Images are at 50x magnification and 400x magnification. Scale bars = 200 *μ*m and 25 *μ*m. Black arrows indicate elastic lamellae. I refers to intima and M refers to media. (b) Neointimal area of carotid arteries within each treatment group. (c) Media area of carotid arteries within each treatment group. (d) Neointima-to-media area ratios of carotid arteries within each treatment group. Data are shown as the mean ± standard deviation. ^##^*P* < 0.01 vs. the control group; ^∗∗^*P* < 0.01 vs. the diabetes mellitus (DM) group. *n* = 6.

Neointima formation in wire-injured carotid arteries of rats was determined using cross sections of carotid arteries by HE staining at 14 days after vascular wire injury and Fc treatment. The neointima area, media area, and neointima-to-media area ratios were analyzed. As shown in Figure 3(a), diabetes significantly increased neointimal hyperplasia compared to the control group. The neointima area (Figure 3(b)) and neointima-to-media area ratios (Figure 3(d)) of injured carotid arteries were significantly increased in rats of the DM group compared to the control group. In contrast, the severe neointimal hyperplasia was attenuated by Fc treatment and the neointima area and neointima-to-media area ratios were decreased in rats of the DM+Fc group compared to the DM group. The media area did not differ significantly among the four groups (Figure 3(c)). Moreover, we found that the neointimal hyperplasia area was inversely associated with the reendothelialization rate.

### 3.3. Fc Increases Beclin 1 Protein of the Intima following Carotid Artery Injury in Diabetic Sprague–Dawley Rats

**Figure 4:**
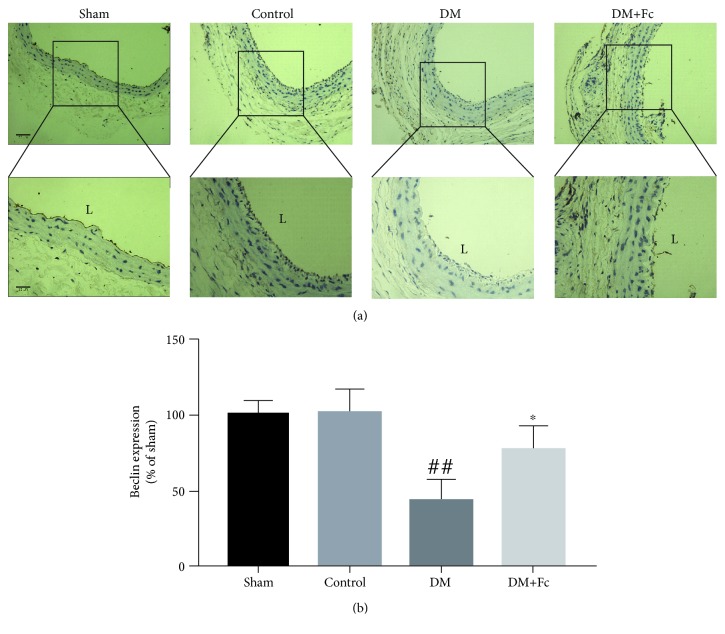
Effects of notoginsenoside Fc (Fc) on Beclin 1 protein of the intima following carotid artery injury. (a) Representative images by immunohistochemistry showing Beclin 1 protein in brown. Beclin 1 protein was found predominantly in the intima. L represents lumen. Images are at 200x and 400x magnification. Scale bars = 50 *μ*m and 25 *μ*m. (b) The number of Beclin 1-positive cells was counted and analyzed statistically in five random high-power fields. Data are shown as the mean ± standard deviation. ^##^*P* < 0.01 vs. the control group; ^∗^*P* < 0.05 vs. the diabetes mellitus (DM) group. *n* = 6.

The effects of Fc on Beclin 1 protein of the intima following carotid artery injury were detected by immunohistochemistry. As shown in Figure 4(a), Beclin 1 protein was found predominantly in the intima. The number of Beclin 1-positive cells was decreased in the DM group rats compared to the control group rats; however, the expression of Beclin 1 of the injured artery was markedly improved by treatment with Fc (Figure 4(b)).

### 3.4. Fc Prevents HG-Induced Autophagy Reduction in Endothelial Cells

**Figure 5:**
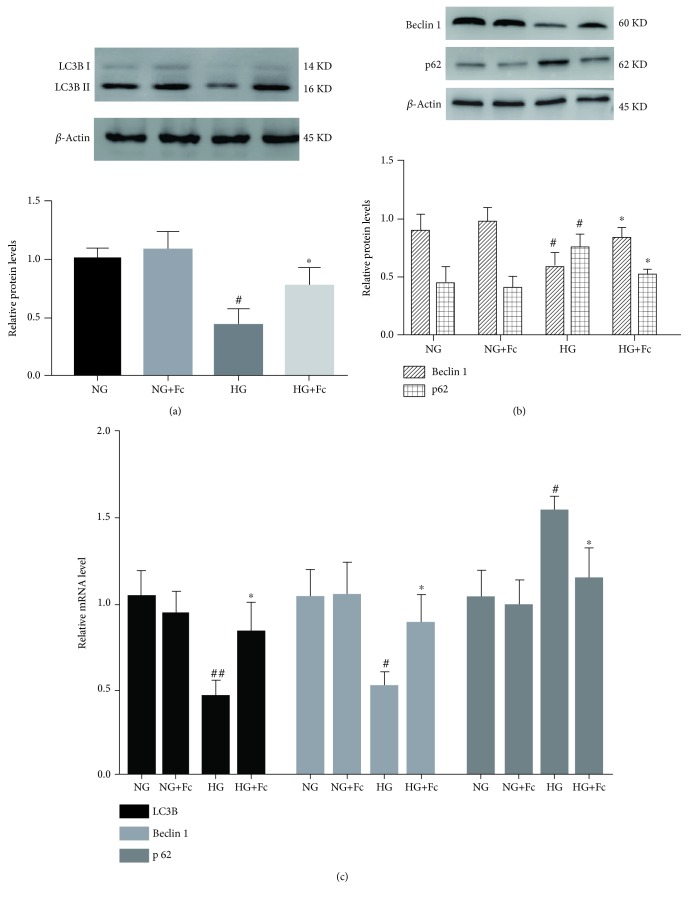
Notoginsenoside Fc (Fc) prevents HG-induced autophagy reduction in rat aortic endothelial cells. (a, b) Expression of LC3B, Beclin 1, and p62 proteins was detected by Western blot analysis. (c) Expression of LC3B, Beclin 1, and p62 mRNA was detected by RT-qPCR. ^#^*P* < 0.05 and ^##^*P* < 0.01 vs. the normal-glucose (NG) group; ^∗^*P* < 0.05 vs. the high-glucose (HG) group. *n* = 3.

**Figure 6:**
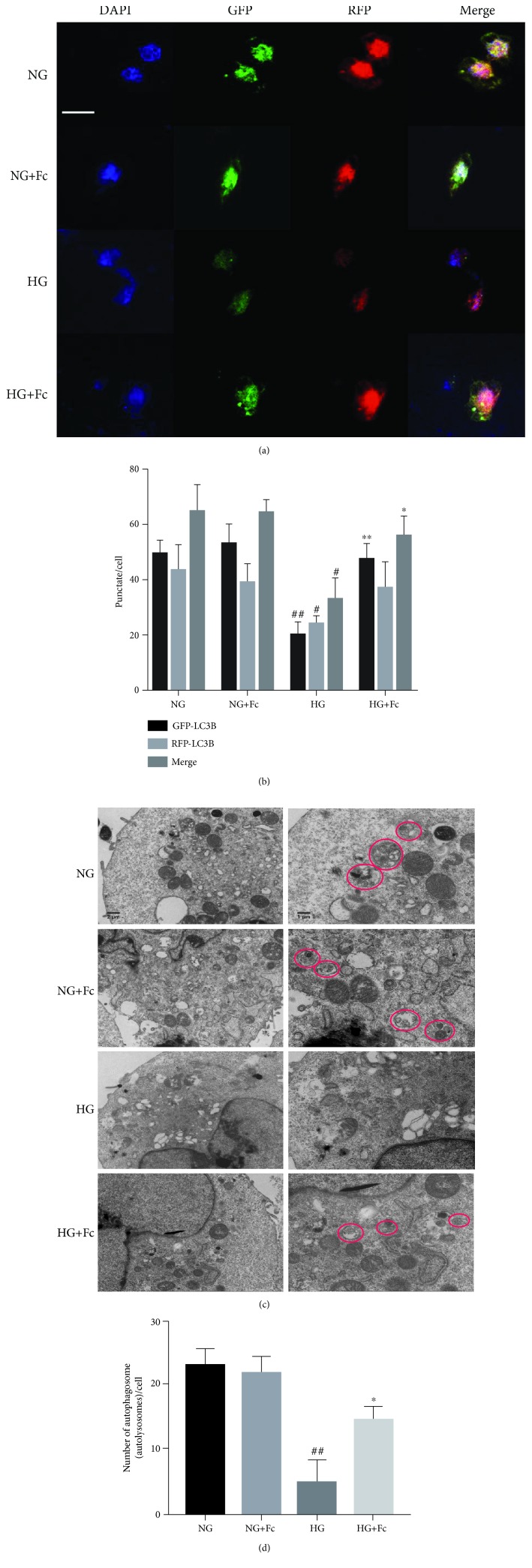
Notoginsenoside Fc (Fc) prevents HG-induced autophagy reduction in rat aortic endothelial cells. (a, b) The mRFP-eGFP-LC3B plasmid was transfected into cells and visualized by confocal microscopy. Representative images show puncta formation in different groups and the quantitative analysis of three types of puncta (8 cells per group). Scale bar = 10 *μ*m. (c, d) Representative images and quantitative analysis of autophagosomes in different groups assessed by electron microscopy (6 cells per group). Black arrows indicate autolysosomes/amphisomes and red arrows indicate autophagosomes. Scale bars = 2 *μ*m and 1 *μ*m. ^#^*P* < 0.05 and ^##^*P* < 0.01 vs. the normal-glucose (NG) group; ^∗^*P* < 0.05 and ^∗∗^*P* < 0.01 vs. the high-glucose (HG) group. *n* = 3.

The effects of Fc on autophagy in RAOECs were first examined by measuring the following autophagy-related markers via Western blot and RT-qPCR analysis: LC3B, Beclin 1, and p62. As shown in Figure 5, HG decreased both protein and mRNA expression of LC3B and Beclin 1 and increased the level of p62. Fc treatment markedly upregulated the expression of LC3B and Beclin 1 and downregulated that of p62 in RAOECs compared to the HG group. To further confirm Fc-induced autophagy in RAOECs, the mRFP-eGFP-LC3B plasmid was transfected into cells and visualized by confocal microscopy. Yellow puncta, reflective of the combination of red fluorescent protein (RFP) and green fluorescent protein (GFP) fluorescence, indicate autophagosomes, which are membrane-bound structures that sequester cellular components, whereas red puncta (RFP only) indicate autolysosomes [24]. Both red and yellow puncta decreased significantly in the HG-treated group compared with the NG group; however, pretreatment with Fc followed by HG increased yellow puncta with no obvious difference in red puncta (Figures 6(a) and 6(b)). Transmission electron microscopy was used to observe autophagosomes when autophagy occurred. Small vacuoles were present in HG-treated RAOECs, but the number increased when the cells were pretreated with Fc (Figures 6(c) and 6(d)).

### 3.5. Fc Promotes RAOEC Proliferation and Migration under HG Conditions via Autophagy

**Figure 7:**
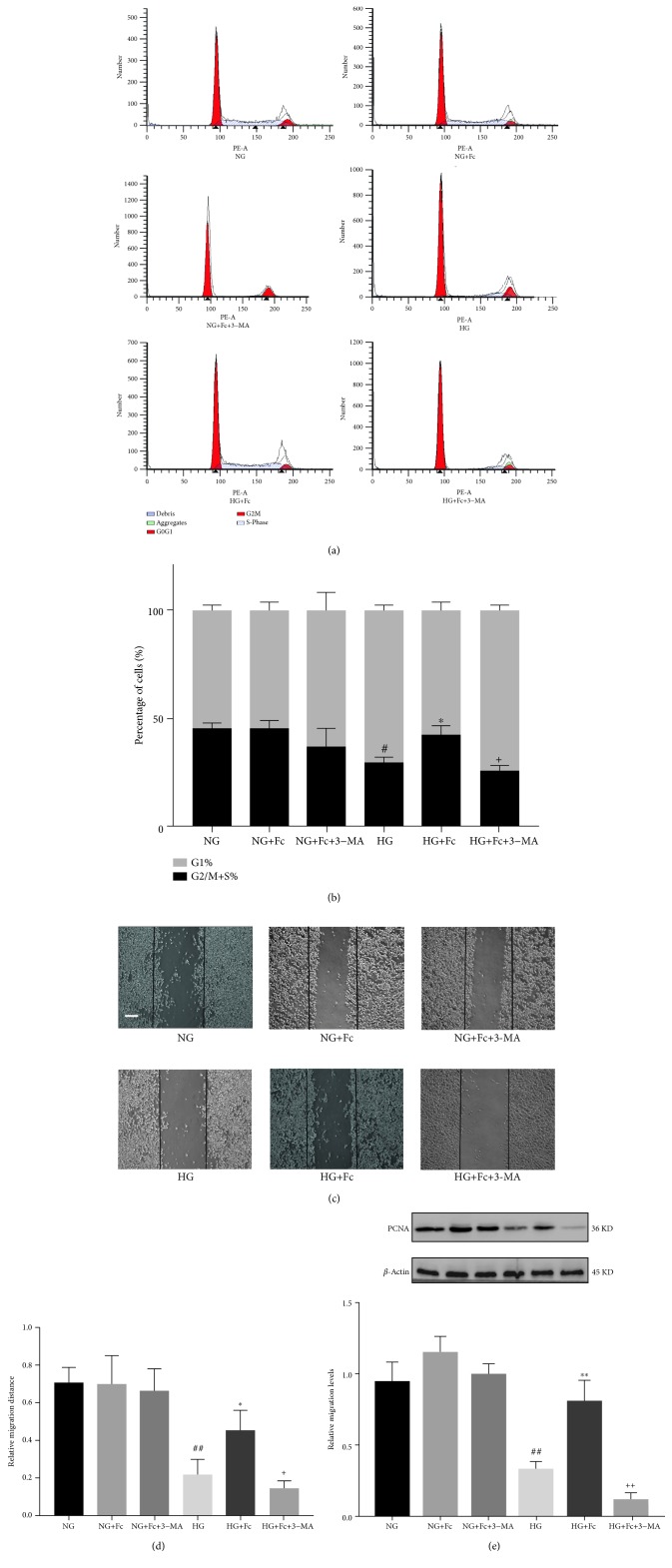
Notoginsenoside Fc (Fc) promotes proliferation and migration via autophagy. (a, b) Cell cycle progression analysis of rat aortic endothelial cells (RAOECs) and G1 arrest rate across treatment groups, as quantified by flow cytometry. (c, d) Representative images of the wound healing assay and wound closure rate in RAOECs across treatment groups. Scale bar = 200 *μ*m. (e) Expression of proliferating cell nuclear antigen (PCNA) protein was detected by Western blot analysis. ^#^*P* < 0.05 vs. the normal-glucose (NG) group; ^##^*P* < 0.01 vs. the NG group; ^∗^*P* < 0.05 vs. the high glucose (HG) group; ^+^*P* < 0.05 vs. the HG+Fc group. *n* = 3.

The effects of Fc-induced autophagy in RAOECs were further confirmed by evaluating Fc-induced cell proliferation and migration after autophagy inhibition. RAOEC proliferation was detected by cell cycle progression analysis (Figures 7(a) and 7(b)). In addition, the protein expression of proliferating cell nuclear antigen (PCNA) was detected by Western blot analysis (Figure 7(e)). More RAOECs were arrested in the G1 phase, and the protein expression of PCNA was lower in HG-treated cells than in NG cells. Fc promoted the proliferation of RAOECs by reversing these changes but decreased following pretreatment with the autophagy inhibitor, 3-MA (5 mM, 2 h). Cell migration was examined using the cell wound healing assay (Figures 7(c) and 7(d)). The migration of RAOECs under HG conditions was significantly reduced compared to NG; cell migration was increased in the HG+Fc group compared to the HG group. However, migration decreased following the addition of 3-MA. Furthermore, cell proliferation and migration did not differ significantly among the NG, NG+Fc, and NG+Fc+3-MA groups.

## 4. Discussion

Hyperglycemia is thought to play an important role in DM by increasing the expression of mitogenic growth factors and inflammatory mediators; during this process, diabetic vascular complications may occur, such as atherosclerosis, diabetic nephropathy, diabetic retinopathy, or myocardial infarction [25]. Interventional surgery has become an important treatment for diabetic vascular complications. Inevitably, it results in vascular endothelium injury, leading to vascular endothelial cell dysfunction [26]. A number of studies have shown that it is of great significance in the prevention of interventional restenosis to form a functional and intact endothelial monolayer early at the injury site, namely, reendothelialization [27–29]. In addition, hyperglycemia in DM plays an essential role in damaging endothelial layer integrity, leading to endothelial dysfunction and injury [25].

Notoginsenoside Fc is the unique ingredient of *P*. *notoginseng* that is commonly used as a platelet aggregation inhibitor or as a blood coagulation inhibitor to prevent or treat thrombotic diseases [10]. Fc has been demonstrated to have a slow elimination from plasma, with a long half-life (22–30 h). When given orally, Fc showed dose-independent pharmacokinetic characteristics and its oral bioavailability was 0.10–0.14% [10]. In addition, Ju et al. indicated that notoginsenoside Fc showed relatively higher exposure in rat plasma after oral administration of saponins from *P. notoginseng* leaves compared with other saponins [7]. Previous studies have reported that improved reendothelialization without platelet adhesion is essential to enhance the long-term patency of blood-contacting devices [30]. Tu et al. found that inhibiting platelet adhesion eventually accelerated the reendothelialization of titanium vascular implants [31]. Another study showed that a bioactive stent surface coating promoted endothelialization while preventing platelet adhesion [32]. Therefore, it is reasonable that Fc compromised endothelial cell repair and accelerated reendothelialization in diabetic rats both *in vitro* and *in vivo*. To our knowledge, this is the first study to demonstrate that Fc accelerates reendothelialization, suggesting that Fc could be developed as a novel therapeutic agent for the prevention and treatment of postinjury restenosis in diabetic vascular complications. The present study focused on reendothelialization, or the repair of endothelial cells after interventional injury. In contrast, in our previous study, which focused on the preventive effect of Fc on endothelial cell injury under HG, Fc alleviated atherosclerosis in diabetic rats [22].

In our *in vivo* experiment, diabetes was established by injection of high doses of STZ. Wire injury of the rat carotid artery was used to simulate vascular injury after intervention. Endothelial recovery, or reendothelialization, after vascular injury (i.e., balloon angioplasty or stent implantation) is clinically relevant to promote vascular healing and to prevent excessive neointimal formation [33]. Meanwhile, studies using animal models have also indicated that delayed endothelial recovery correlates with increased neointimal hyperplasia after vascular injury [34, 35]. Evans blue staining is a reliable way to detect reendothelialization [36]. Our results confirmed that Fc treatment following rat carotid artery injury accelerated endothelial repair and alleviated neointimal hyperplasia in DM rats. These data support the beneficial effects of the use of Fc to prevent postinjury restenosis in diabetes.

Next, we investigated how Fc affected reendothelialization and ultimately neointimal formation. Autophagy plays a crucial role in maintaining endothelial integrity and promoting endothelial repair [17]. Du et al. reported that autophagy may be an important promoter of angiogenesis, as both pharmacological inhibition and genetic inhibition of autophagy significantly impair migration in endothelial cells [37]. In addition, we found that Fc attenuated HG-induced vascular endothelial cell injury via upregulation of PPAR-*γ* in diabetic rats. Furthermore, PPAR-*γ* mediated autophagy and Yuan et al. indicated that PPAR-*γ* signaling inhibited cardiac hypertrophy via activation of autophagy [38]. Beclin 1 levels were detected *in vivo* by immunohistochemistry. Beclin 1 is a mammalian autophagy gene that is central to autophagy regulation [17]. It was found predominantly in the intima of the vasculature. The results indicated that autophagy of injured carotid arteries was decreased in the DM group compared to the control group; however, autophagy was markedly improved following Fc treatment. Three different approaches were used *in vitro* to determine the role of autophagy in RAOECs. During autophagy, the cytosolic form of LC3B-I is converted into LC3B-II, a phosphatidylethanolamine-conjugated form, to promote autophagosome formation, and the amount of LC3B-II is a commonly used indicator of autophagy [39]. In this study, protein and mRNA levels of LC3B-II and Beclin 1 were determined by Western blot and RT-qPCR analysis. Both were decreased under HG conditions but increased following treatment with Fc. In addition, p62, an autophagic substrate, whose level inversely correlates with autophagic flux [40], was downregulated after Fc treatment. When cells are transfected with LC3B constructed with the GFP and RFP plasmid (mRFP-eGFP-LC3B), autophagosome formation and autophagy flux can be visualized via yellow and red puncta [41]. Our data suggested a decrease in the number of autophagosomes and autolysosomes in the HG group but an increase in autophagosomes only in the HG+Fc group. Autophagy was simultaneously observed by transmission electron microscopy, further confirming the increased number of autophagosomes induced by Fc under HG conditions. Together, these findings support Fc-dependent prevention of HG-induced autophagy reduction in endothelial cells and that the target may be prior to the formation of autophagosomes.

The disruption of endothelial monolayer integrity induced by hyperglycemia in DM can be alleviated by the proliferation and migration of neighboring endothelial cells [42]. We confirmed that Fc could promote RAOEC proliferation and migration under HG conditions. To future explore the link between Fc-induced autophagy and proliferation, we used a low concentration of 3-MA, a synthetic intermediate, and cell-permeable autophagic sequestration blocker. We found that Fc-induced proliferation and migration were reversed when autophagy was inhibited, suggesting that Fc promotes RAOEC proliferation and migration under HG conditions via autophagy.

There were limitations to this study. First, we used only one dose of Fc in the *in vivo* study. Therefore, the dose-dependent effects of Fc *in vivo* should be examined in a future study. Second, the blood concentration and bioavailability of Fc were not measured in the animals and these should also be examined. Therefore, the applicability of Fc to early endothelial injury and restenosis following intervention in diabetic vascular complications needs to be further investigated.

## 5. Conclusions

In conclusion, notoginsenoside Fc is a relatively new and slightly studied saponin isolated from *P. notoginseng*. Our study is the first to report that Fc can promote endothelial cell proliferation and migration under HG conditions *in vitro*, ultimately accelerating reendothelialization in the peripheral vascular system of diabetic rats *in vivo* by promoting autophagy. These data suggest that Fc exerts a potential therapeutic benefit for early endothelial injury and restenosis following intervention in diabetes-associated peripheral vascular diseases.

## Data Availability

All data used to support the findings of this study are included within the article.
